# Secondary Adrenal Insufficiency: An Overlooked Cause of Hyponatremia

**DOI:** 10.14740/jocmr2041w

**Published:** 2015-02-09

**Authors:** Naureen Jessani, Waqas Jehangir, Daisy Behman, Abdalla Yousif, Ira J. Spiler

**Affiliations:** a Raritan Bay Medical Center, Perth Amboy, NJ, USA

**Keywords:** Depression, Hyponatremia, SIADH

## Abstract

Failure to thrive in an elderly patient is often attributed to depression, especially when a patient does not have any chronic diseases or if there is no apparent medical reason to justify poor appetite, cachexia and generalized weakness. Hyponatremia often occurs in such patients and a thorough evaluation as to its etiology should be sought before committing to a premature diagnosis, which at the time may seem more plausible. We report a patient who presented with depression, weight loss and persistent hyponatremia, evaluation of which revealed the cause to be due to secondary adrenal insufficiency, which when treated, resulted in resolution of the symptom complex. Therefore, in our case report, we elucidate the importance of pursuing further evaluation to rule out adrenal insufficiency as a medical cause of depression, especially in the presence of hyponatremia, which is often overlooked and is generally attributed to dehydration in the setting of failure to thrive or SIADH in patients who are on psychotropic medications.

## Introduction

Depression is a known cause of failure to thrive in elderly patients, which not only needs prompt diagnosis and treatment but also mandates a thorough workup to rule out medical causes. Several medical conditions are associated with failure to thrive and depression, including hypothyroidism, hyperthyroidism, diabetes mellitus, vitamin B12 deficiency, malignancy, electrolyte abnormalities, substance abuse and medications. Persistent hyponatremia in a depressed patient should direct clinicians to revisit the case in quest of delineating its cause. SIADH is often cited as a cause of hyponatremia in patients who have been on anti-depressants or anti-psychotics but often other causes of hyponatremia are overlooked. Secondary adrenal insufficiency can present with failure to thrive, psychiatric symptoms and hyponatremia; in fact, in most cases, hyponatremia is the only manifestation of secondary adrenal insufficiency and if diagnosed timely, can lead to its resolution.

## Case Report

A 70-year-old female from Germany, recently widowed, was admitted under psychiatric service for major depression. She was brought in by her son for weight loss, dehydration and inactivity. Her medical history was remarkable for essential hypertension, which was well controlled on medications (no diuretics), and depression, for which she had multiple hospitalizations and had intermittently taken anti-depressants. She denied ever being on steroids for any reason. Her review of systems was essentially unremarkable. She was hydrated and was started on perphenazine, which she had been periodically on in past years.

**Figure 1:**
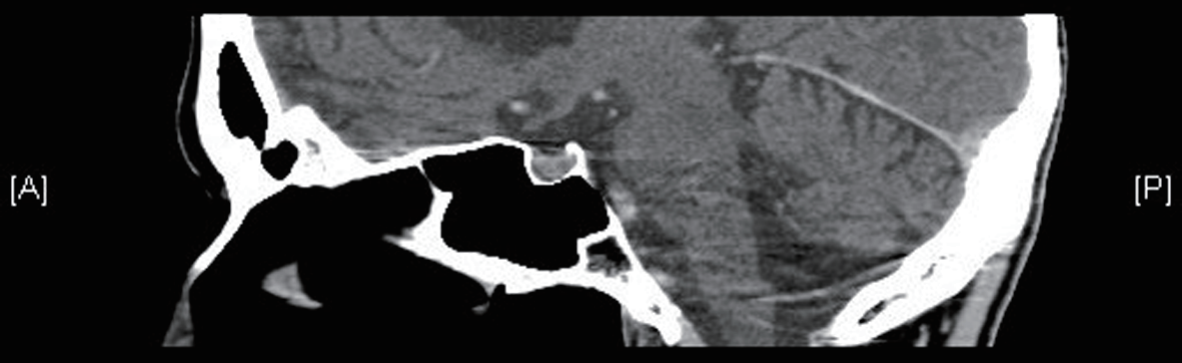
Hypo-enhancing focus within the right anterior sella consistent with pituitary microadenoma.

She continued to be withdrawn and delusional and refused to tend to activities of daily living for several weeks and her hyponatremia persisted, at which time, an endocrine evaluation was initiated. A comprehensive workup was done, which revealed a relatively low cortisol level with a blunted response on cosyntropin stimulation and an undetectable ACTH (Table 1, 2). Although her albumin level was a little low but it was not low enough to necessitate measurement of free cortisol level. Further endocrine workup is shown in Table 3. CT of head with contrast was done to rule out a mass lesion or an empty sella. CT scan revealed a 3 mm hypo-dense, relatively hypo-enhancing focus within the right anterior sella consistent with pituitary microadenoma (Fig. 1).

**Table 1:** Initial Serum and Urine Electrolytes

	Serum	Urine
Osmolality	273 mOsm/kg	614 mOsm/kg
Sodium	129 mmol/L	106 mmol/L
Potassium	4 mmol/L	57 mmol/L
Chloride	98 mmol/L	91 mmol/L
Bicarbonate	22 mmol/L	
Albumin	3.1 g/dL	

**Table 2:** Cosyntropin Stimulation Test

Test	Baseline	30 min	60 min
ACTH	< 1.1 pg/mL		
Cortisol	7.1 μg/dL	9.90 μg/dL	12.30 μg/dL
Aldosterone	1.20 ng/dL		

**Table 3:** Hormone Assays

Hormone	Level	Normal range
FSH	11.70	25.8 - 134.8 mIU/mL
LH	2.10	7.7 - 58.5 mIU/mL
TSH	0.52	0.27 - 4.2 mIU/mL
Free T4	1.40	0.93 - 1.7 ng/dL
Prolactin	102.30	4.8 - 23.3 ng/mL

Patient was placed on stress dose of hydrocortisone which was rapidly tapered to physiologic replacement. Her appetite and energy level improved within a day or two of starting steroids. She became more interactive, gained weight and her hyponatremia resolved (Table 4). Patient was transferred to a psychiatric facility for further care.

**Table 4:** Treatment Outcome

	Pre-treatment	Post-treatment
Sodium	129 mmol/L	133 mmol/L

## Discussion

Elderly patients are often brought to the emergency department because of failure to thrive. The Institute of Medicine defined failure to thrive late in life as a syndrome manifested by weight loss greater than 5% of baseline, decreased appetite, poor nutrition, and inactivity, often accompanied by dehydration, depressive symptoms, impaired immune function, and low cholesterol levels [1]. Since these patients have poor oral intake, hyponatremia is often attributed to dehydration, which is expected to correct on rehydration, and if persists, should instigate further workup.

Depression can be a cause and a consequence of failure to thrive. Therefore, screening for depression is necessary for all patients who exhibit characteristics of failure to thrive [2]. Failure to thrive is also known to be seen in patients with adrenal insufficiency, and more often than not, these patients are found to be depressed. In fact, chronic adrenal insufficiency can present with psychiatric symptoms, which include organic brain syndrome (5-20%), memory impairment, depression (20-40%) and psychosis (20-40%) [3]. How it causes these manifestations is not known though. Most of these symptoms disappear within a few days of initiation of glucocorticoid therapy. Since our patient had psychiatric manifestations; we inferred that it could be partly related to adrenal insufficiency. Basic metabolic workup in patients with depression includes CBC, complete metabolic panel, TSH, vitamin B12 level and RPR. Our case report emphasizes the significance of doing a cortisol level in the evaluation of depressed patients, especially if they have refractory hyponatremia. Cortisol level is central to the stress response and usually is elevated in acutely depressed patients. Our patient had low morning cortisol, which prompted us to do further workup but even a normal cortisol level in acutely depressed patients cannot reliably exclude adrenal insufficiency and warrants cosyntropin stimulation test.

Hyponatremia is often seen in patients with adrenal insufficiency, which is caused by an inappropriate increase in vasopressin secretion/action due to cortisol deficiency [4] and inability to excrete free water. Cortisol deficiency results in increased hypothalamic secretion of corticotropin releasing hormone (CRH), an ADH secretagogue. Cortisol feeds back negatively on CRH and ACTH, an inhibitory effect that is removed with adrenal insufficiency. In addition, cortisol appears to directly suppress ADH secretion. Thus, ADH levels increase when plasma cortisol levels are low. Hypothyroidism also causes hyponatremia utilizing the same mechanism of action [5]. Adrenal insufficiency can be primary or secondary. Primary adrenal insufficiency manifests as hyperpigmentation and hyperkalemia whereas secondary adrenal insufficiency presents as failure to thrive, psychiatric symptoms and hyponatremia. All patients with euvolemic hyponatremia need further evaluation but in the presence of an alternative apparent explanation for hyponatremia, which in this case was dehydration, failure to thrive and psychotropic medications, adrenal insufficiency can go undiagnosed if not thought about. Clinicians should be mindful of various causes of hyponatremia, some of which can be easily overlooked [6].

Treatment goal includes hormone supplementation, dose adjustments during concurrent ailments and periodic monitoring for systemic side effects. Glucocorticoid administration leads to return of sodium level into the normal range within a few days. Literature lacks specific guidelines about increasing the dose in times of mental stress in a patient with known adrenal insufficiency. Cortisol is as critical for emotional wellbeing as it is for physical wellbeing. Increasing the dose of glucocorticoid in times of major depression definitely leads to improved energy levels and psychological wellbeing.

Patients with secondary adrenal insufficiency should be evaluated for other pituitary hormone deficiencies, which could coexist. Post-menopausal patients have high gonadotropin levels unlike our patient who had low gonadotropin levels, which could either be secondary to hypopituitarism or due to cachexia or could be explained by elevated prolactin level. An elevated prolactin level could have been due to anti-psychotic medications or pituitary micro-adenoma but no treatment was deemed necessary as patient was post-menopausal and hyperprolactinemia was asymptomatic. The better explanation of elevated prolactin can be obtained when the levels are repeated periodically and classically off antipsychotics.

### Conclusion

Our case report highlights several key points. The foremost among them is that hyponatremia secondary to adrenal insufficiency is not so uncommon and high level of suspicion should be maintained while evaluating a patient with chronic or persistent hyponatremia, who present with psychiatric manifestations. SIADH is a common cause of hyponatremia in depressed and psychotic patients but is a diagnosis of exclusion. Clinicians should be cognizant of the fact that adrenal insufficiency does not only cause hyponatremia but also can present as failure to thrive, depression and psychosis. It is important to obtain adequate laboratory evaluation at the time of presentation so that a diagnosis can be reached promptly followed by a timely treatment.
